# Pathogenesis of Alcohol-Associated Fatty Liver: Lessons From Transgenic Mice

**DOI:** 10.3389/fphys.2022.940974

**Published:** 2022-07-05

**Authors:** Afroza Ferdouse, Robin D. Clugston

**Affiliations:** Department of Physiology, University of Alberta, Edmonton, AB, Canada

**Keywords:** alcohol, alcohol-associated fatty liver disease, steatosis, fatty acid uptake, *de novo* lipogenesis, mitochondrial beta-oxidation, triglyceride metabolism

## Abstract

Alcohol-associated liver disease (ALD) is a major public health issue that significantly contributes to human morbidity and mortality, with no FDA-approved therapeutic intervention available. The health burden of ALD has worsened during the COVID-19 pandemic, which has been associated with a spike in alcohol abuse, and a subsequent increase in hospitalization rates for ALD. A key knowledge gap that underlies the lack of novel therapies for ALD is a need to better understand the pathogenic mechanisms that contribute to ALD initiation, particularly with respect to hepatic lipid accumulation and the development of fatty liver, which is the first step in the ALD spectrum. The goal of this review is to evaluate the existing literature to gain insight into the pathogenesis of alcohol-associated fatty liver, and to synthesize alcohol’s known effects on hepatic lipid metabolism. To achieve this goal, we specifically focus on studies from transgenic mouse models of ALD, allowing for a genetic dissection of alcohol’s effects, and integrate these findings with our current understanding of ALD pathogenesis. Existing studies using transgenic mouse models of ALD have revealed roles for specific genes involved in hepatic lipid metabolic pathways including fatty acid uptake, mitochondrial β-oxidation, *de novo* lipogenesis, triglyceride metabolism, and lipid droplet formation. In addition to reviewing this literature, we conclude by identifying current gaps in our understanding of how alcohol abuse impairs hepatic lipid metabolism and identify future directions to address these gaps. In summary, transgenic mice provide a powerful tool to understand alcohol’s effect on hepatic lipid metabolism and highlight that alcohol abuse has diverse effects that contribute to the development of alcohol-associated fatty liver disease.

## Introduction

Chronic excess alcohol consumption is a major public health issue, and one of the leading causes of liver disease (Sherlock and Dooley, 2008; Bruha et al., 2012; WHO, 2018). Excess alcohol consumption is associated with the development of alcohol-associated liver disease (ALD), which includes a well-described spectrum of disease ranging from hepatic fat accumulation (steatosis) to cirrhosis and hepatocellular carcinoma (Miller et al., 2011; Ceni et al., 2014). It is estimated that 1 in 3 people drink alcohol around the world (GBD 2016 Alcohol Collaborators, 2018). While drinking patterns vary from country to country, the overall health burden of alcohol use is high, and ranks in the top ten risk factors for death and disability-adjusted life-years (DALYs) GBD 2016 Alcohol Collaborators (2018). Alcohol consumption and abuse are prevalent in North America. In the United States, 85.6% of adults reported drinking alcohol at some point in life, and 25.8% drink heavily (Redlich et al., 1998). In Canada, 78.2% of the total population reported drinking alcohol in the past year and 19.1% reported heavy drinking (Canadian Center on Substance Use and Addiction, 2019). According to the World Health Organization (WHO), global alcohol consumption including that in the United States and Canada is predicted to further increase by 2025 (WHO, 2018). While alcohol has always historically been associated with ALD, this health burden has worsened during the COVID-19 pandemic, which has been associated with a spike in alcohol abuse, and a subsequent increase in hospitalization rates for ALD (Shaheen et al., 2021; Sohal et al., 2021; Stockwell et al., 2021).

Strikingly, there are no FDA-approved therapies for the treatment of ALD (Osna et al., 2017; Singal and Shah, 2019). This represents an unmet need to treat patients with ALD and has its origins in an incomplete understanding of ALD pathogenesis. The initial phase of ALD is the development of hepatic steatosis, which develops in approximately 90%–100% of heavy drinkers (consuming ≥60 g/day of alcohol) and predisposes these individuals to more severe liver disease (Crabb, 1999; Mann et al., 2003; Singal and Shah, 2019). Alcohol is known to have wide-ranging effects on hepatic lipid metabolism leading to the accumulation of hepatic fat, as recently reviewed by others (You and Arteel 2019; Jeon and Carr 2020; Hyun et al., 2021).

The goal of this review is to evaluate the existing literature to gain insight into the pathogenesis of alcohol-associated fatty liver, with a specific emphasis on information garnered from transgenic mouse models (summarized in Table 1). This approach allows for a genetic dissection of alcohol’s effects on hepatic lipid metabolism, which we integrate with our current understanding of ALD pathogenesis in humans. In addition to reviewing this literature, we aim to discuss the limitations of working with transgenic mouse models, identify current gaps in our understanding of how alcohol abuse impairs hepatic lipid metabolism, and identify future directions to address these gaps.

**TABLE 1:** Summary of study design and key findings from transgenic mouse models of ALD.

Author, year (PMID)	Gene (background)	Sex	Alcohol feeding protocol	ALD phenotype	Putative mechanism
Clugston et al. (2014) (24280415)	*Cd36* ^*−/−*^(C57BL/6)	Male	LDeC, 5.1% (v/v) alcohol, 6 weeks	Alleviated	Impaired FA uptake (?), decreased DNL
Nakajima et al. (2004) (15382117)	*Pparα* ^*−/−*^ (Sv/129)	Male	LDeC, 4.0% (v/v) alcohol, 6 months	Worsened	Impaired mitochondrial β-oxidation
Ji et al. (2006) (16879892)	*Srebp1c* ^*−/−*^ (mixed)	Male	Intragastric infusion, 4.4% (v/v), 4 weeks	Alleviated	Decreased DNL
Lounis et al. (2016) (27477676)	*Scd1* ^*−/−*^ (C57BL/6N)	Male	LDeC, 5.0% (v/v) alcohol, 10 days plus binge (5 g/kg)	Alleviated	Decreased DNL
Zhang et al. (2016) (27062444)	PPARγ∆-Hep (mixed)	Male	LDeC, 5.6% (v/v) alcohol, 8 weeks	Alleviated	Decreased DNL, decreased TG synthesis
Huang et al. (2018) (30192394)	*Dgat1* ^*−/−*^ (C57BL/6)	Male	LDeC, 5.1% (v/v) alcohol, 6 weeks	Alleviated	Decreased TG synthesis
Xu et al. (2016) (27075303)	*Ces1* ^*−/−*^ (C57BL/6J)	Not mentioned	LDeC, 5.0% (v/v) alcohol, 10 days plus binge (3 g/kg)	Worsened	Increased DNL, impaired mitochondrial β-oxidation
Carr et al. (2014) (24831094)	*Plin2* ^*−/−*^ (C57BL/6J)	Male	LDeC, 2.71% (v/of calories from alcohol, 6 weeks	Alleviated	Impaired LD formation

## Alcohol’s Effects on Hepatic Lipid Metabolism

**FIGURE 1:**
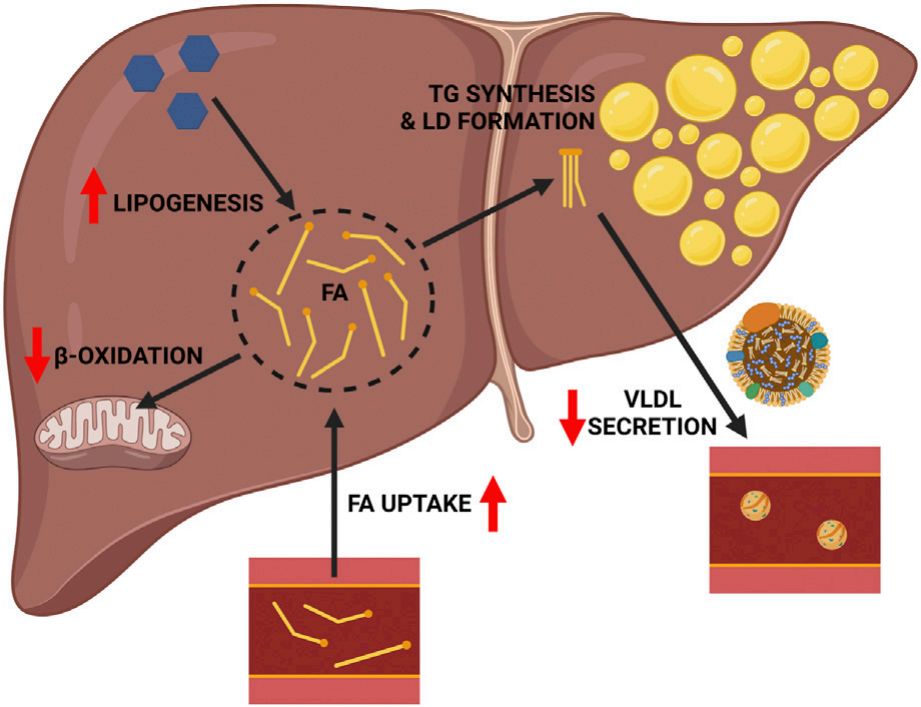
General mechanisms linking chronic alcohol consumption with hepatic lipid accumulation. Chronic alcohol consumption impacts hepatic lipid metabolism and drives the accumulation of hepatic triglycerides in several ways, including: 1) increased FA uptake, 2) decreased mitochondrial β-oxidation, and 3) increased hepatic *de novo* lipogenesis. These effects increase the hepatic FA pool available for esterification into triglyceride and lipid droplet formation. This effect is further compounded by 4) decreased VLDL triglyceride secretion. Red arrows indicate the detrimental effects of alcohol. Image created with BioRender.com.

The liver is of central importance in whole-body lipid metabolism in mammals (Alves-Bezerra and Cohen, 2017). The liver also serves as the primary organ for alcohol detoxification, the metabolic burden of which directly and indirectly affects the complex and interconnected pathways of hepatic lipid metabolism, precipitating the development of hepatic steatosis. As reviewed elsewhere (You and Arteel, 2019; Jeon and Carr, 2020), alcohol impacts multiple aspects of hepatic lipid metabolism including increased hepatic fatty acid (FA) uptake, increased hepatic *de novo* lipogenesis (DNL), decreased mitochondrial β-oxidation, and decreased very low density lipoprotein (VLDL) secretion, the net effect of which is increased hepatic lipid accumulation (Figure 1). In terms of increased hepatic FA uptake, chronic alcohol consumption increases adipose tissue lipolysis, which leads to an increase in circulatory free FA available for hepatic uptake (Kang et al., 2007; Zhong et al., 2012; Wei et al., 2013). Regarding DNL, the predominant mechanism is alcohol-induced activation of sterol regulatory element binding protein 1 (SREBP1c), a transcriptional regulator of multiple genes in the DNL pathway, including Acetyl CoA Carboxylase (*Acc*), FA Synthase (*Fasn*), and Stearoyl CoA Desaturase 1 (*Scd1*) (You et al., 2002). Alcohol’s ability to impair mitochondrial β-oxidation is multifactorial, but is primarily driven by two factors: first, an increase in the hepatic NADH:NAD + ratio secondary to alcohol oxidation, favoring this process over FA oxidation (Lieber, 1988); and second, by decreasing Peroxisome Proliferation Activator Receptor-α (PPARα) activity, a transcriptional regulator of several genes associated with mitochondrial β-oxidation (Nanji et al., 2004). Alcohol’s ability to impair VLDL secretion is linked to impaired lipidation of Apo-B, resulting in reduced VLDL particle formation and export of hepatic lipids (Sugimoto et al., 2002; Kharbanda et al., 2012). As noted above, these broad effects of alcohol on hepatic lipid metabolism leads to an increase in the hepatic FA pool, which can be esterified and stored in lipids droplets as triglycerides (TGs). The following sections will further explore each of these pathways, and the importance of using transgenic mice to understand their contribution to ALD.

## Hepatic FA Uptake

Chronic alcohol consumption induces the lipolysis of TGs stored in white adipose tissue (WAT), which enter the circulation and can be taken up by the liver (Figure 1). Insulin is the major hormone that supresses adipose lipolysis (Stumvoll et al., 2001). Chronic ethanol feeding induces insulin resistance that markedly impairs the anti-lipolytic effects of insulin in WAT, enhancing TG breakdown to release free FA into the circulation (Kang et al., 2007). Circulating free FAs derived from adipose lipolysis are directly taken up by hepatocytes with the help of FA transport proteins (FATPs) or FA translocase/CD36 (Kang et al., 2007; Jeon and Carr, 2020). Among the multiple FATP family members, FATP2 and FATP5 are highly expressed in the liver (Stahl et al., 2001). CD36 expression is normally low in the healthy liver but its expression is induced in the alcohol exposed liver (Ge et al., 2010; Clugston et al., 2011; Ronis et al., 2011; Zhong et al., 2012). This observation led to the hypothesis that alcohol-induced upregulation of CD36 promotes the uptake of circulating FAs, contributing to the development of alcohol-associated fatty liver. Clugston et al. (2014) tested this hypothesis in *Cd36* ^*−/−*^ mice consuming a low-fat/high carbohydrate Lieber-DeCarli (LDeC) liquid diet with 5.1% (v/v) alcohol for 6 weeks. Importantly, the blood alcohol concentration was not different between alcohol fed wild-type (WT) and *Cd36* ^*−/−*^ mice, indicating no effect of CD36 deficiency on ethanol metabolism. Histological and biochemical analysis of hepatic TGs clearly demonstrated that CD36 deficiency had a protective role in the development of alcohol-associated steatosis. However, follow-up analysis indicated that this protective effect was not linked to altered FA uptake, and no compensatory changes in the expression level of *Fatp2* or *Fatp5* were observed in *Cd36* ^*−/−*^ mice. Interestingly, expression studies examining the DNL pathway and *in vivo* kinetic studies indicated that the rate of hepatic DNL was reduced in *Cd36* ^*−/−*^ mice. This data suggested that in alcohol consuming WT mice, a higher rate of DNL provided additional FA for TG synthesis, a finding that was underscored by a significant increase in *Dgat2* expression only in alcohol-fed WT mice, a TG synthesizing enzyme that is known to be coupled to DNL (Wurie et al., 2012). Thus, genotype-specific changes in DNL were the major contributor to the protective effect of CD36 deficiency. Indeed, this link between CD36 and DNL was recently elucidated and linked to CD36’s ability to regulate SREBP1c, a key transcriptional regulator of the DNL pathway (Zeng et al., 2022).

In summary, alcohol feeding studies in *Cd36* ^*−/−*^ mice indicate that CD36 is important in the pathogenesis of ALD, although a direct effect on hepatic FA uptake was ruled out (Clugston et al., 2014). It is important to highlight that this study was conducted in mice consuming the low-fat/high carbohydrate formulation of LDeC liquid diets. As discussed below, this lipogenic diet may favor hepatic DNL as a driver of hepatic lipid accumulation as opposed to increased hepatic FA uptake, which might be more associated with the high-fat/low carbohydrate formulation of the LDeC liquid diet. The pathogenesis of ALD in *Cd36* ^*−/−*^ mice consuming the high-fat/low carbohydrate formulation of the LDeC liquid diet has not been reported. The existing literature supports a role for increased hepatic uptake in the pathogenesis of ALD, which is coupled to increased WAT lipolysis (Kang et al., 2007). The importance of this pathway was recently highlighted when it was shown that modulating FA disposal *via* brown adipose tissue impacted the severity of ALD (Shen et al., 2019). The question remains, what is the primary mediator of hepatic FA uptake in the alcohol-exposed liver, and can this be targeted to ameliorate ALD? While FATP2 and FATP5 are possible candidates their role in ALD has not been directly studied and requires further investigation.

## Mitochondrial β-Oxidation

Alcohol’s ability to impair mitochondrial β-oxidation is another mechanism that can contribute to channeling of hepatic FAs toward TGs formation and the development of hepatic steatosis. As reviewed elsewhere (Jeon and Carr, 2020), there are different mechanisms through which alcohol inhibits mitochondrial β-oxidation, although one of the primary ways is thought to be by decreasing PPARα activity (Wan et al., 1995; Galli et al., 2001; Fischer et al., 2003; Jeon and Carr, 2020). PPARα is an important transcriptional regulator of several genes associated with mitochondrial β-oxidation (Yu et al., 2003), and free FAs and their derivatives serve as ligands for PPARα and activate PPARα signaling to stimulate mitochondrial β-oxidation of hepatic FAs (Keller et al., 1993; Forman et al., 1997).

The central role of PPARα signaling in hepatic lipid metabolism and data linking ALD with altered PPARα activity has led to extensive investigations into the role of PPARα in the pathogenesis of ALD. Indeed, earlier studies in mice showed that ethanol feeding impaired the activity of PPARα resulting in decreased expression of PPARα target genes related to FA β-oxidation (Fischer et al., 2003). Moreover, treatment with a PPARα agonist (Wy14,643) restored the activation of PPARα and target gene expression, thereby increasing FA β-oxidation in alcohol fed mice and preventing steatosis formation (Fischer et al., 2003). Similarly, downregulated PPARα responsive genes have also been reported in alcohol fed rats, which corresponded to increased steatosis and liver injury, and that PPARα activation with Clofibrate restores the expression of PPARα regulated genes, and reduces steatosis severity and markers of ALD (Nanji et al., 2004).

With growing evidence linking altered PPARα activity with ALD, studies leveraging transgenic mice were conducted. In 2004, Nakajima et al. reported that ALD is worsened in *Ppara* ^*−/−*^ mice (Nakajima et al., 2004). These authors fed WT and *Ppara* ^*−/−*^ mice 4% (v/v) ethanol containing LDeC liquid diets for 6 months. At the end of the study period liver injury was markedly more pronounced in alcohol consuming *Ppara* ^*−/−*^ mice, including worsened hepatomegaly and evidence of severe hepatocyte damage, inflammation, and fibrosis that was not observed in WT mice. The authors concluded that loss of PPARα exacerbated ALD through several mechanisms including acetaldehyde accumulation, impaired antioxidant capacity of the liver, and potentiated proinflammatory signaling *via* NF-κB. Regarding steatosis, baseline levels of hepatic TGs were higher in the *Ppara* ^*−/−*^ mice, which is in accord with the phenotype of these mice (Kersten et al., 1999); however, the authors noted that while hepatic TG levels increased to a similar extent after 6 months of alcohol feeding, analysis at earlier time points showed that hepatic TG accumulation was higher in the *Ppara* ^*−/−*^ mice compared to WT. This led the authors to suggest that PPARα’s ability to dispose of hepatic FA, *via* mitochondrial β-oxidation, is another important mechanism through which PPARα deficiency exacerbates ALD, and that this pathway may be a significant contributor to the development of steatohepatitis.

With the establishment of *Ppara* ^*−/−*^ mice as a model of severe ALD, this transgenic mouse has been further used to study the pathogenesis of ALD. The importance of oxidative stress in ALD was highlighted in alcohol consuming *Ppara* ^*−/−*^ mice treated with the antioxidant polyenephosphatidylcholine (PPC) (Okiyama et al., 2009). This study showed that PPC treatment ameliorated severe ALD in *Ppara* ^*−/−*^ mice, including reduced markers of hepatocyte damage and death, inflammation, and fibrosis; however, PPC treatment did not improve markers of hepatic steatosis. The beneficial effect of PPC treatment was linked to decreased expression of enzymes associated with the generation of reactive oxygen species (i.e., CYP2E1), and improved markers of hepatic oxidative stress. The link between alcohol’s effects on PPARα and hepatic lipid metabolism has also been further explored in *Ppara* ^*−/−*^ mice (Li et al., 2014). Li et al. (2014) confirmed that alcohol consumption is associated with a downregulation of PPARα activity, decreased mitochondrial β-oxidation, and the development of steatosis. The role of PPARα was emphasized by the exacerbation of ALD in *Ppara* ^*−/−*^ mice, as evidenced by increased markers of TG accumulation, inflammation, and fibrosis. The authors of this study concluded that PPARα played a protective role in ALD, that was primarily mediated through enhanced mitochondrial function, including mitochondrial β-oxidation.

As a regulator of mitochondrial β-oxidation, it is clear that altered PPARα activity is an important factor in the pathogenesis of alcohol-associated fatty liver, although it is equally clear that PPARα deficiency exacerbates ALD through multiple mechanisms in addition to impaired FA disposal (Nakajima et al., 2004). In their paper, Nakajima and colleagues highlighted that alcohol fed *Ppar*α^*−/−*^ mice showed many pathogenic hallmarks of ethanol toxicity that mirror human cases of advanced ALD (Nakajima et al., 2004). While data from human ALD is limited, there is evidence of reduced functional PPARα in the human liver, which may partly explain why humans are more susceptible to ethanol-induced liver toxicity than rodents (Tugwood et al., 1996; Hertz and Bar-Tana, 1998; Palmer et al., 1998). Interestingly, although PPARα agonism has shown to ameliorate ALD in rodent models (Fischer et al., 2003; Nanji et al., 2004), this work has not thus far been translated into humans, despite its potential as a novel therapeutic in ALD (Li et al., 2014). Interestingly, while targeting PPARα may have a beneficial effect in the liver, there may be additional benefits to targeting PPARα in the context of human ALD, including reduced ethanol consumption (Barson et al., 2009; Blednov et al., 2015). In closing, while genetic manipulation of PPARα provides key evidence for the importance of FA oxidation in the pathogenesis of ALD, the broad effects of this transcription factor limit the interpretation of this data. To our knowledge, genetic manipulation of other key factors involved in FA oxidation have not been reported.

## *De Novo* Lipogenesis

The process by which liver synthesizes FA from non-lipid precursor molecules is called DNL. Catabolism of non-lipid precursors such as glucose, and even ethanol, can generate pyruvate to contribute to the TCA cycle, which is subsequently converted to Acetyl CoA and used for the synthesis of FAs (Siler et al., 1999; Yamashita et al., 2001; Ameer et al., 2014; Charidemou et al., 2019). Key transcriptional regulators of DNL include SREBP1c and ChREBP (carbohydrate responsive element binding protein). These transcription factors modulate the expression of key enzymes in the DNL pathway including *Acc*, *Fasn*, and *Scd1* (Denechaud et al., 2008). As introduced above, it is thought that upregulation of the DNL pathway is one of the contributors to the development of hepatic steatosis in ALD. Indeed, the literature includes reports of increased *Srebp1c*, *Acc*, *Scd1*, and *Fasn* expression in the alcohol exposed liver (Foufelle and Ferre, 2002; Ntambi et al., 2002; You et al., 2002; MacDonald et al., 2008; Huang et al., 2013; Lounis et al., 2016). As discussed below, key insight into the role of altered DNL in ALD comes from alcohol feeding studies in *Srebp1c* ^*−/−*^ and *Scd1* ^*−/−*^ mice.

### Evidence From *Srebp1c* ^*−/−*^ Mice

As a transcriptional regulator of multiple key genes in the DNL pathway, analysis of SREBP1c’s contribution to the pathogenesis of ALD is important. Indeed, it has been shown that both acute and chronic alcohol consumption can induce SREBP1c protein and its transcript in mice/rodents (You et al., 2002; Yin et al., 2007). Ji et al. (2006) used *Srebp1c* ^*−/−*^ mice to test the hypothesis that ALD is dependent on SREBP1c. These authors performed their study in WT and *Srebp1c* ^*−/−*^ mice on a C57BL/6 background fed alcohol 4.4% (v/v) for 4 weeks by intragastric infusion. Consistent with the hypothesized role of SREBP1c, *Srebp1c* ^*−/−*^ mice were largely protected from ALD, including ameliorated hepatomegaly, hepatic TG accumulation and inflammation. Mechanistically, it was shown that while alcohol consumption induced markers of hepatic DNL (e.g., ACC), this effect was absent in alcohol fed *Srebp1c* ^*−/−*^ mice, leading to the conclusion that the lipogenic pathway was not activated in these mice, protecting them from hepatic lipid accumulation (Ji et al., 2006). Mechanistically, the authors linked the alcohol associated induction of SREBP1c with endoplasmic reticulum stress secondary to alcohol’s effect on homocysteine metabolism, and showed that treatment with betaine, which prevents homocysteine accumulation, prevented SREBP1c induction and ALD (Ji et al., 2006).

### Evidence From *Scd1* ^*−/−*^ Mice

SCD1 is a δ-9 FA desaturase which catalyses the formation of monounsaturated fatty acids and is an important contributor to hepatic DNL (Cohen et al., 2002). Several studies have shown that SCD1 deficient mice are protected from the development of non-alcoholic fatty liver disease (NAFLD) (Ntambi et al., 2002; MacDonald et al., 2008). In the context of alcohol abuse, it has been shown that both chronic and binge alcohol consumption increases *Scd1* expression in mice (Huang et al., 2013; Zhang et al., 2015). These observations lead to the hypothesis that SCD1 deficiency might protect against ALD, which was tested by Lounis et al. (2016). These authors compared WT and *Scd1* ^*−/−*^ mice using the chronic-binge model of ALD, which consists of mice consuming LDeC diets with 5% (v/v) alcohol for 10 days, followed by a single binge dose of alcohol (5 g/kg body weight) (Bertola et al., 2013). Strikingly, alcohol-fed *Scd1* ^*−/−*^ mice were strongly protected against ALD in comparison to their WT controls. This phenotype included a normalization of hepatic lipid TG levels in alcohol-fed *Scd1* ^*−/−*^, normalized serum liver enzymes (AST and ALT), and decreased markers of hepatic inflammation. Mechanistically, the authors showed a strong induction of the DNL pathway in alcohol-fed WT mice, including increased expression of *Srebp1c*, *Acc*, *Fasn*, and *Scd1*, which was normalized in *Scd1* ^*−/−*^ mice, leading to the conclusion that SCD1 deficiency prevented the upregulation of the DNL pathway and prevented ALD. Interestingly, while alcohol consumption was associated with decreased *Ppar*α and *Cpt1a* expression in WT mice, baseline levels of these genes in *Scd1* ^*−/−*^ mice were elevated and unaffected by alcohol consumption, suggesting that genotype-specific effects on mitochondrial β-oxidation may have also contributed to the protective effect of SCD1 deficiency. Mechanistically, increased mitochondrial β-oxidation may also protect SCD1 deficient mice from NAFLD (Ntambi et al., 2002). Taken together, analysis of alcohol consuming *Scd1* ^*−/−*^ mice provides compelling evidence for the involvement of the DNL pathway in the pathogenesis of ALD.

Taken together, there is strong evidence from *Srebp1c* ^*−/−*^ and *Scd1* ^*−/−*^ mice supporting a role for increased DNL as a significant contributor to hepatic lipid accumulation in ALD. Interestingly, as discussed above the DNL pathway can also be modulated through ChREBP, which links hepatic carbohydrate and lipid metabolism, and it has been repeatedly shown that alcohol can also induce ChREBP (Wada et al., 2008; Liangpunsakul et al., 2013; Marmier et al., 2015; Gao et al., 2016; Zhang et al., 2017; Xue et al., 2021). Thus, while there is clear evidence that alcohol induced activation of DNL *via* SREBP1c contributes to ALD, a role for ChREBP is also likely, although to our knowledge *Chrebp* ^*−/−*^ mice have not been studied in the context of ALD.

## Triglyceride Metabolism and Lipid Droplet Formation

TGs are the major type of neutral lipid that accumulates in the cytosolic lipid droplets of the steatotic liver in ALD (Carr and Ahima, 2016; Jeon and Carr, 2020). This section describes insight gained from transgenic mice targeting different aspects of TG metabolism in the context of ALD, including the transcriptional regulation of hepatic lipogenesis and TG synthesis by peroxisome proliferator-activated receptor gamma (PPARγ), TG synthesis by DGAT1, TG hydrolysis by CES1, and lipid droplet homeostasis by PLIN2. The phenotypes described below emphasize the important insight that can be gained into the pathogenesis of ALD through the study of transgenic mouse models.

### Evidence From *Ppar*γ Transgenic Mice

PPARγ belongs to the Type II nuclear hormone receptor superfamily and has pleiotropic effects on hepatic lipid metabolism (Tomita et al., 2004; Pettinelli and Videla, 2011; Zhang et al., 2016). Elevation of hepatic PPARγ expression is a common feature of steatosis in ALD and NAFLD (Schadinger et al., 2005; Pettinelli and Videla, 2011; Yu et al., 2016). Whereas it has also been shown that PPARγ agonists can alleviate liver damage in ALD (Enomoto et al., 2003; Tomita et al., 2004). These studies highlight an important role for hepatic PPARγ signaling in ALD. While whole body deletion of *Ppar*γ is embryonic lethal (Barak et al., 1999), the role of PPARγ in ALD has been studied in mice using hepatocyte specific PPARγ knock-down (PPARγ∆-Hep) (Zhang et al., 2016). Zhang et al. (2016) generated PPARγ∆-Hep mice by crossing Albumin-Cre transgenic mice and PPARγ^flox/flox^ mice, and experimentally these mice were provided with LDeC diets containing 5.6% (v/v) alcohol for 8 weeks. In agreement with a role for PPARγ signaling in ALD, PPARγ∆-Hep mice were protected from alcohol-induced hepatic steatosis, with a blunted increase in liver TGs in alcohol fed mice. Follow-up studies suggest that this effect was primarily driven by effects on hepatic lipogenesis, with no evidence to support a modulatory effect on FA uptake and oxidation, or VLDL secretion. Regarding DNL, the alcohol-associated induction of SREBP1c and *Fasn* was blunted in PPARγ∆-Hep mice, although surprisingly *Acc* induction was unaffected. Regarding TG synthesis, alcohol’s ability to induce both *Dgat1* and *Dgat2* was abolished in PPARγ∆-Hep mice. These data were interpreted to suggest that PPARγ signaling is an important contributor to ALD, which is associated with its ability to promote hepatic lipid accumulation primarily through its effects on DNL and TG synthesis (Zhang et al., 2016).

### Evidence From *Dgat1* ^*−/−*^ Mice

DGAT1 and DGAT2 catalyze the final step in TG synthesis and both can have a role in hepatic TG accumulation and steatosis. Studies of partitioning in hepatic TG synthesis suggest that DGAT1 has a substrate preference for exogenous FAs, whereas DGAT2 prefers endogenous FAs produced by DNL (Clugston et al., 2014; Li et al., 2015a). In the context of ALD, both *Dgat1* and *Dgat2* have been shown to be induced in the alcohol-exposed liver leading to hepatic steatosis (Wang et al., 2010; Clugston et al., 2011; Clugston et al., 2014; Zhang et al., 2016). In order to test the hypothesis that DGAT1 contributes to ALD, Huang et al. (2018) conducted a series of alcohol feeding studies in *Dgat1* ^*−/−*^ mice on a C57BL/6 background. These authors provided experimental mice with 5.1% (v/v) alcohol containing LDeC diets for 6 weeks, uniquely using both the high-fat/low carbohydrate formulation of the LDeC diets, and the low-fat/high-carbohydrate formulation. Interestingly, this study revealed that DGAT1 deficiency protects against the development of alcoholic steatosis in mice when alcohol was provided with a high-fat/low carbohydrate, but not low-fat/high-carbohydrate diet, as evidenced by hepatic Oil Red O staining and biochemical measurement of hepatic TGs. This observation led to the conclusion that DGAT1 mediates hepatic steatosis in the context of a high-fat/low carbohydrate diet, presumably coupling with increased exogenous FA supply to the liver, whereas in the context of a low-fat/high-carbohydrate diet that favors DNL, DGAT2 predominates. As discussed above, these differential roles of DGAT1 and DGAT2 are consistent with the existing data (Clugston et al., 2014; Li et al., 2015b; Huang et al., 2018). While this study supports the importance of TG synthesis by DGAT1 in ALD, studies into *Dgat2* transgenic mice are limited because *Dgat2* ^*−/−*^ mice are not viable (Stone et al., 2004).

### Evidence From *Ces1* ^*−/−*^ Mice

DGAT1 and DGAT2 are the two enzymes responsible for TG synthesis in the liver, yet there are numerous enzymes that hydrolyse TGs in this organ that have specific physiological functions (Gilham and Lehner, 2004). One such TG hydrolase is Carboxylesterase 1 (CES1), which is a serine esterase that is able to hydrolyze TGs and cholesteryl esters (Quiroga et al., 2012; Xu et al., 2014). There are limited studies on the differential expression of CES1 in ALD, although its expression has been reported to be reduced in alcohol-associated steatohepatitis, and in alcohol consuming mice (Xu et al., 2016). Based on this observation, Xu et al. (2016) tested whether CES1 has a role in the pathogenesis of ALD, showing that *Ces1* ^*−/−*^ mice have worsened ALD. Using *Ces1* ^*−/−*^ mice on a C57BL/6J background, experimental animals were fed 5% (v/v) alcohol containing LDeC diet for 10 days followed by an ethanol binge (3 g/kg body weight). *Ces1* ^*−/−*^ mice were reported to have worsened liver damage and hepatic inflammation in response to alcohol feeding, including elevated liver enzymes, increased expression of inflammatory genes, worsened markers of oxidative stress, and mitochondrial dysfunction. While the authors reported increased hepatic free FA levels in alcohol consuming *Ces1* ^*−/−*^ mice, TG levels were not different, which the authors attributed to possible differences in intestinal alcohol metabolism or absorption. To circumvent this limitation the authors did show increased TG accumulation in alcohol fed mice with hepatic knockdown of *Ces1*, which was linked with increased markers of DNL (Xu et al., 2016). While we have framed these results in the context of TG metabolism, it is important to recognize that loss of CES1 may directly impact hepatic acetaldehyde metabolism, with resultant effects on oxidative stress and metabolism (Xu et al., 2016). Taken together, hepatic CES1 has a protective role in alcohol induced hepatic steatosis and its deficiency worsens hepatic steatosis by inducing DNL and liver injury in ALD.

### Evidence From *Plin2* ^*−/−*^ Mice

LDs are enveloped by a phospholipid monolayer associated with LD proteins, in the liver the most abundant LD associated protein is Perilipin 2 (PLIN2) (Itabe et al., 2017). Moreover, it has been shown that PLIN2 expression levels are increased in mice and rat models of ALD (Mak et al., 2008; Straub et al., 2008; Orlicky et al., 2011), and is thought to be a marker of hepatic steatosis development (Carr et al., 2014). Carr et al. (2014) studied *Plin2* ^*−/−*^ mice to test the hypothesis that PLIN2 deficiency would prevent hepatic lipid accumulation in alcohol consuming mice. Using LDeC liquid diets with up to 2.7% (v/v) of alcohol for 6 weeks, this group revealed that PLIN2 deficiency protects against the development of alcohol associated steatosis (Carr et al., 2014). Similar to WT control and *Plin2* ^*−/−*^ control mice, alcohol fed *Plin2* ^*−/−*^ mice showed no histologic evidence of alcoholic steatosis, and hepatic TG levels were not elevated by alcohol consumption. Ceramides derived from the sphingomyelin from LD membranes are known to be elevated in ALD (Liangpunsakul et al., 2010; Longato et al., 2012), and a temporal relationship with PLIN2 upregulation and ceramide accumulation has been reported in ALD (Carr et al., 2013). While alcohol feeding increased C22 and C24 species of ceramide in WT mice, this effect is blunted in alcohol fed *Plin2* ^*−/−*^ mice, indicating that PLIN2 also has a role in alcohol induced hepatic ceramide accumulation. Interestingly, PLIN2 deficiency had a beneficial effect on insulin sensitivity, suggesting that loss of PLIN2 was able to protect against ALD-associated hepatic insulin resistance, an effect that may be secondary to impaired hepatic lipid accumulation in these mice.

Taken together and consistent with alcohol’s diverse effects on hepatic lipid metabolism and the central importance of TG metabolism in the development of steatosis, these studies reveal critical regulators of hepatic lipid accumulation in ALD. This includes the protective effect of deficient lipogenic signaling (*Ppara* ^*−/−*^ mice), TG synthesis (*Dgat1* ^*−/−*^), and lipid droplet formation (*Plin2* ^*−/−*^) in ALD, as well as the worsening effect of impaired TG hydrolysis (*Ces1* ^*−/−*^).

## Concluding Remarks

**FIGURE 2:**
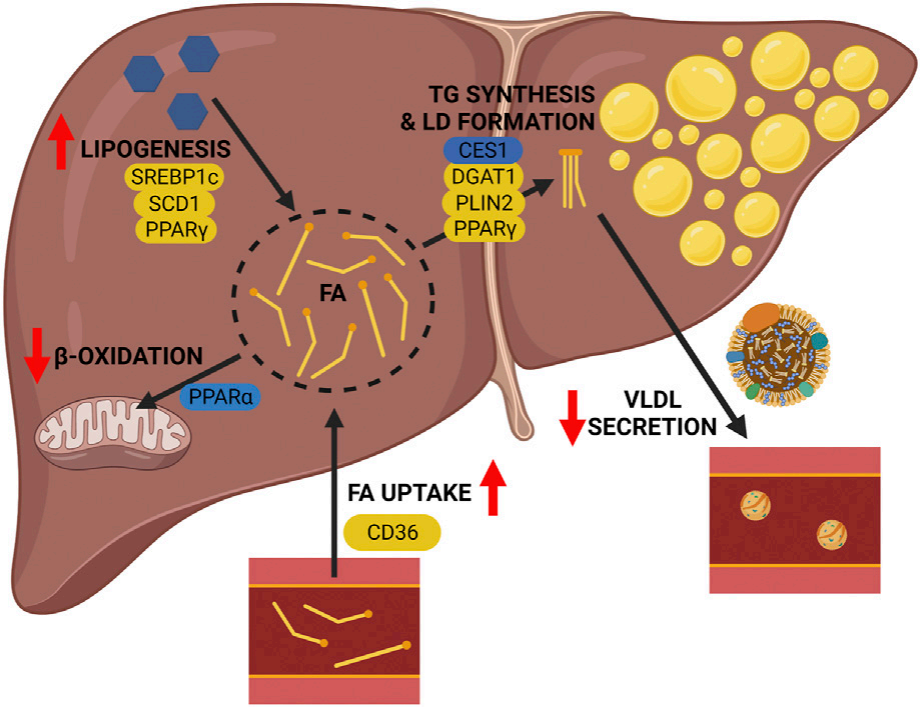
Pathogenesis of alcohol-associated fatty liver: lessons from transgenic mice. Genetic deletion of key genes in lipid metabolic pathways of the liver reveal their contribution to the pathogenesis of ALD. The following pathway-associated genes were found to be important contributors to ALD, including *Cd36* (hepatic FA uptake), *Pparα* (mitochondrial β-oxidation), *De novo* lipogenesis (*Srebp1c*, *Scd1*, and *Ppar*γ), and triglyceride metabolism and lipid droplet formation (*Ces1*, *Dgat1*, *Plin2*, and *Ppar*γ). Genetic deletion of genes shown in yellow alleviate the effects of alcohol, and genetic deletion of genes shown in blue worsen the effects of alcohol. Image created with BioRender.com.

Transgenic mouse models of ALD can help elucidate the mechanisms by which alcohol consumption contributes to alcohol associated fatty liver. It is widely acknowledged that alcohol has multiple effects on hepatic lipid metabolism (Figure 1), and through the use of transgenic mouse models we can identify specific key regulators associated with different lipid metabolic pathways that contribute to hepatic lipid accumulation in ALD (summarized in Figure 2; Table 1). As reviewed herein, gene knock outs in hepatic FA uptake (*Cd36* ^*−/−*^), DNL (*Srebp1c* ^*−/−*^ and *Scd1* ^*−/−*^), and TG metabolism (*Ppar*γ^*−/−*^, *Dgat1* ^*−/−*^, and *Plin2* ^*−/−*^) protect against ALD, whereas gene knocks out in mitochondrial β-oxidation (*Ppara* ^*−/−*^) and TG hydrolysis (*Ces1* ^*−/−*^) exacerbate ALD. Interestingly, although it is accepted that alcohol abuse impacts multiple aspects of hepatic lipid accumulation causing steatosis, the relative importance of these different pathways is unclear. Others have speculated that impaired mitochondrial β-oxidation is the most significant contributor to alcohol associated steatosis (Jeon and Carr 2020; Hyun et al., 2021). While there is strong evidence to support this conclusion, the current review shows that genetic ablation of key components in other metabolic pathways is sufficient to ameliorate ALD independently of mitochondrial β-oxidation. Further study is required to understand the relative contribution of different lipid metabolic pathways to the development of alcohol associated steatosis. It is encouraging that genetic ablation of single genes in different metabolic pathways can ameliorate hepatic lipid accumulation in ALD, as this broadens the potential therapeutic targets to treat ALD in its early stages.

### Limitations of Transgenic Mouse Models

While transgenic mouse models are a powerful tool to study ALD, there are inherent weaknesses in their application and gaps in our knowledge remain. At the level of experimental design, it is important to acknowledge that all of the studies reviewed in this manuscript that report sex, only use male mice (Table 1). The clinical presentation of ALD is different in men and women, thus it is incumbent upon the research community to include female mice in their study of ALD pathogenesis (Han et al., 2021). Another important aspect of experimental design is the choice of alcohol feeding protocol. The majority of studies reviewed here utilize LDeC liquid diets, with variations in duration and the amount of alcohol (Table 1). These models consistently produce hepatic lipid accumulation in response to alcohol, although it is acknowledged that they are limited in their ability to produce severe ALD [e.g., steatohepatitis and fibrosis (Bertola et al., 2013)]. Interestingly, the macronutrient composition of the LDeC liquid diets may have an impact on the predominant pathways leading to hepatic lipid accumulation, with differences dependent on whether FAs are produced endogenously (e.g., *via* DNL), or exogenously (e.g., *via* FA uptake), representing an important consideration when designing studies and translating their results (Li et al., 2015a; Huang et al., 2018). Perhaps one of the most significant limitations in the majority of studies included in this manuscript are the use of global knockouts of genes of interest. While these studies are focused on hepatic lipid metabolism, it should be acknowledged that whole body ablation of specific genes may effect whole body lipid metabolism that might also indirectly impact hepatic lipid metabolism. The use of liver specific knockouts would address this limitation and has the added advantage of circumventing embryonic lethality of specific gene knockouts. For example, Zhang et al. (2016) used liver specific ablation of *Ppar*γ to circumvent the lethality of global knockout of this gene. This approach could be used to further study alcohol’s effects on hepatic lipid metabolism, for example while *Dgat2* ^*−/−*^ mice are not viable, a liver specific knockout has been described that could be used to dissect the role of this TG synthesizing enzyme in ALD (Stone et al., 2004; Gluchowski et al., 2019). Finally, while transgenic mouse models are a powerful tool to study ALD pathogenesis, there is an obvious need to translate these studies into humans if a clinical benefit is to be realized. While limited human data is available, there are clear parallels between the effect of alcohol on the mouse and human liver. Indeed, similar to the data generated from mice there are human studies implicating alcohol-induced changes in hepatic FA uptake, mitochondrial β-oxidation, and DNL (Blomstrand et al., 1973; Siler et al., 1999; Rachakonda et al., 2014). Nevertheless, data on molecular mediators of these effects in humans is limited, validating the use of rodent models to gain detailed insight into ALD pathogenesis.

### Knowledge Gaps and Future Directions

While the limitations discussed above are important considerations in our interpretation of transgenic mouse data and their potential translation, we also have to acknowledge that significant gaps remain in our understanding of alcohol’s effects on hepatic lipid metabolism that could benefit from genetic dissection. As indicated above, each of the major lipid metabolic pathways could be further probed using transgenic mice. For example, given the apparent interaction between CD36 deficiency and DNL (Clugston et al., 2014; Zeng et al., 2022), studies in FATP2 or 5 deficient mice may help better understand the importance of FA uptake in the pathogenesis of ALD. Similarly, genetic targeting of a critical regulator of mitochondria β-oxidation (e.g., *Cpt1a*) would directly probe this pathway separate from the broad effects of *Ppara* ^*−/−*^ mice. Use of *Srebp1c* ^*−/−*^ and *Scd1* ^*−/−*^ mice have provided good coverage of the DNL pathway, but there is also an opportunity to study *Chrebp* ^*−/−*^ mice to better understand alcohol’s effect on this regulator of DNL. Moreover, the effect of alcohol on VLDL secretion has been neglected. While genetic targets exist to study this pathway (e.g., *ApoB* or *Mttp*) alcohol feeding studies have not been conducted in transgenic mice targeting these genes.

### Conclusion

Transgenic mouse models have been effectively used to study ALD and understand its molecular pathogenesis; however, as discussed above, there is significant opportunity to leverage genetically engineered mouse models targeting different pathways of hepatic lipid accumulation that will provide further insight into ALD. Ultimately, there is consensus in the field that further study of alcohol’s effect on hepatic lipid metabolism is warranted to guide the development of much needed treatments for ALD (Jeon and Carr, 2020; Hyun et al., 2021).

## References

[B1] Alves-Bezerra M. Cohen D. E. (2017). Triglyceride Metabolism in the Liver. Compr. Physiol. 8 (1), 1–8. 10.1002/cphy.c170012 29357123 PMC6376873

[B2] Ameer F. Scandiuzzi L. Hasnain S. Kalbacher H. Zaidi N. (2014). De Novo lipogenesis in Health and Disease. Metabolism 63 (7), 895–902. 10.1016/j.metabol.2014.04.003 24814684

[B3] Barak Y. Nelson M. C. Ong E. S. Jones Y. Z. Ruiz-Lozano P. Chien K. R. (1999). PPARγ Is Required for Placental, Cardiac, and Adipose Tissue Development. Mol. Cell. 4 (4), 585–595. 10.1016/s1097-2765(00)80209-9 10549290

[B4] Barson J. R. Karatayev O. Chang G.-Q. Johnson D. F. Bocarsly M. E. Hoebel B. G. (2009). Positive Relationship between Dietary Fat, Ethanol Intake, Triglycerides, and Hypothalamic Peptides: Counteraction by Lipid-Lowering Drugs. Alcohol 43 (6), 433–441. 10.1016/j.alcohol.2009.07.003 19801273 PMC2758659

[B5] Bertola A. Mathews S. Ki S. H. Wang H. Gao B. (2013). Mouse Model of Chronic and Binge Ethanol Feeding (The NIAAA Model). Nat. Protoc. 8 (3), 627–637. 10.1038/nprot.2013.032 23449255 PMC3788579

[B6] Blednov Y. A. Benavidez J. M. Black M. Ferguson L. B. Schoenhard G. L. Goate A. M. (2015). Peroxisome Proliferator-Activated Receptorsαandγare Linked with Alcohol Consumption in Mice and Withdrawal and Dependence in Humans. Alcohol Clin. Exp. Res. 39 (1), 136–145. 10.1111/acer.12610 25516156 PMC4308472

[B7] Blomstrand R. Kager L. Lantto O. (1973). Studies on the Ethanol-Induced Decrease of Fatty Acid Oxidation in Rat and Human Liver Slices. Life Sci. 13 (8), 1131–1141. 10.1016/0024-3205(73)90380-9 4762602

[B8] Bruha R. Dvorak K. Petrtyl J. (2012). Alcoholic Liver Disease. Wjh 4 (3), 81–90. 10.4254/wjh.v4.i3.81 22489260 PMC3321494

[B9] Canadian Center on Substance Use and Addiction (2019). Alcohol. AvaliableAt: https://www.ccsa.ca/sites/default/files/2019-09/CCSA-Canadian-Drug-Summary-Alcohol-2019-en.pdf (Accessed December 12, 2021).

[B10] Carr R. M. Ahima R. S. (2016). Pathophysiology of Lipid Droplet Proteins in Liver Diseases. Exp. Cell. Res. 340 (2), 187–192. 10.1016/j.yexcr.2015.10.021 26515554 PMC4744586

[B11] Carr R. M. Dhir R. Yin X. Agarwal B. Ahima R. S. (2013). Temporal Effects of Ethanol Consumption on Energy Homeostasis, Hepatic Steatosis, and Insulin Sensitivity in Mice. Alcohol Clin. Exp. Res. 37 (7), 1091–1099. 10.1111/acer.12075 23398239 PMC3657580

[B12] Carr R. M. Peralta G. Yin X. Ahima R. S. (2014). Absence of Perilipin 2 Prevents Hepatic Steatosis, Glucose Intolerance and Ceramide Accumulation in Alcohol-Fed Mice. PLoS One 9 (5), e97118. 10.1371/journal.pone.0097118 24831094 PMC4022498

[B13] Ceni E. Mello T. Galli A. (2014). Pathogenesis of Alcoholic Liver Disease: Role of Oxidative Metabolism. Wjg 20 (47), 17756–17772. 10.3748/wjg.v20.i47.17756 25548474 PMC4273126

[B14] Charidemou E. Ashmore T. Li X. McNally B. D. West J. A. Liggi S. (2019). A Randomized 3-way Crossover Study Indicates that High-Protein Feeding Induces De Novo Lipogenesis in Healthy Humans. JCI Insight 4 (12), e124819. 10.1172/jci.insight.124819 PMC6629161 31145699

[B15] Clugston R. D. Jiang H. Lee M. X. Piantedosi R. Yuen J. J. Ramakrishnan R. (2011). Altered Hepatic Lipid Metabolism in C57BL/6 Mice Fed Alcohol: a Targeted Lipidomic and Gene Expression Study. J. Lipid Res. 52 (11), 2021–2031. 10.1194/jlr.m017368 21856784 PMC3196234

[B16] Clugston R. D. Yuen J. J. Hu Y. Abumrad N. A. Berk P. D. Goldberg I. J. (2014). CD36-deficient Mice Are Resistant to Alcohol- and High-Carbohydrate-Induced Hepatic Steatosis. J. Lipid Res. 55 (2), 239–246. 10.1194/jlr.m041863 24280415 PMC3886662

[B17] Cohen P. Miyazaki M. Socci N. D. Hagge-Greenberg A. Liedtke W. Soukas A. A. (2002). Role for Stearoyl-CoA Desaturase-1 in Leptin-Mediated Weight Loss. Science 297 (5579), 240–243. 10.1126/science.1071527 12114623

[B18] Crabb D. W. (1999). Pathogenesis of Alcoholic Liver Disease. Newer Mechanisms of Injury. Keio J. Med. 48 (4), 184–188. 10.2302/kjm.48.184 10638142

[B19] Denechaud P.-D. Dentin R. Girard J. Postic C. (2008). Role of ChREBP in Hepatic Steatosis and Insulin Resistance. FEBS Lett. 582 (1), 68–73. 10.1016/j.febslet.2007.07.084 17716660

[B20] Enomoto N. Takei Y. Hirose M. Konno A. Shibuya T. Matsuyama S. (2003). Prevention of Ethanol-Induced Liver Injury in Rats by an Agonist of Peroxisome Proliferator-Activated Receptor-γ, Pioglitazone. J. Pharmacol. Exp. Ther. 306 (3), 846–854. 10.1124/jpet.102.047217 12805475

[B21] Fischer M. You M. Matsumoto M. Crabb D. W. (2003). Peroxisome Proliferator-Activated Receptor α (PPARα) Agonist Treatment Reverses PPARα Dysfunction and Abnormalities in Hepatic Lipid Metabolism in Ethanol-Fed Mice. J. Biol. Chem. 278 (30), 27997–28004. 10.1074/jbc.m302140200 12791698

[B22] Forman B. M. Chen J. Evans R. M. (1997). Hypolipidemic Drugs, Polyunsaturated Fatty Acids, and Eicosanoids Are Ligands for Peroxisome Proliferator-Activated Receptors α and δ. Proc. Natl. Acad. Sci. U.S.A. 94 (9), 4312–4317. 10.1073/pnas.94.9.4312 9113986 PMC20719

[B23] Foufelle F. Ferré P. (2002). New Perspectives in the Regulation of Hepatic Glycolytic and Lipogenic Genes by Insulin and Glucose: a Role for the Transcription Factor Sterol Regulatory Element Binding Protein-1c. Biochem. J. 366 (Pt 2), 377–391. 10.1042/BJ20020430 12061893 PMC1222807

[B24] Galli A. Pinaire J. Fischer M. Dorris R. Crabb D. W. (2001). The Transcriptional and DNA Binding Activity of Peroxisome Proliferator-Activated Receptor α Is Inhibited by Ethanol Metabolism. J. Biol. Chem. 276 (1), 68–75. 10.1074/jbc.m008791200 11022051

[B25] Gao L. Shan W. Zeng W. Hu Y. Wang G. Tian X. (2016). Carnosic Acid Alleviates Chronic Alcoholic Liver Injury by Regulating the SIRT1/ChREBP and SIRT1/p66shc Pathways in Rats. Mol. Nutr. Food Res. 60 (9), 1902–1911. 10.1002/mnfr.201500878 27125489

[B26] GBD 2016 Alcohol Collaborators (2018). Alcohol Use and Burden for 195 Countries and Territories, 1990-2016: a Systematic Analysis for the Global Burden of Disease Study 2016. Lancet 392 (10152), 1015–1035. 10.1016/S0140-6736(18)31310-2 30146330 PMC6148333

[B27] Ge F. Zhou S. Hu C. Lobdell H. Berk P. D. (2010). Insulin- and Leptin-Regulated Fatty Acid Uptake Plays a Key Causal Role in Hepatic Steatosis in Mice with Intact Leptin Signaling but Not Inob/obordb/dbmice. Am. J. Physiology-Gastrointestinal Liver Physiology 299 (4), G855–G866. 10.1152/ajpgi.00434.2009 PMC2957339 20595619

[B28] Gilham D. Lehner R. (2004). The Physiological Role of Triacylglycerol Hydrolase in Lipid Metabolism. Rev. Endocr. Metab. Disord. 5 (4), 303–309. 10.1023/b:remd.0000045101.42431.c7 15486462

[B29] Gluchowski N. L. Gabriel K. R. Chitraju C. Bronson R. T. Mejhert N. Boland S. (2019). Hepatocyte Deletion of Triglyceride‐Synthesis Enzyme Acyl CoA: Diacylglycerol Acyltransferase 2 Reduces Steatosis without Increasing Inflammation or Fibrosis in Mice. Hepatology 70 (6), 1972–1985. 10.1002/hep.30765 31081165 PMC6893913

[B30] Han S. Yang Z. Zhang T. Ma J. Chandler K. Liangpunsakul S. (2021). Epidemiology of Alcohol-Associated Liver Disease. Clin. Liver Dis. 25 (3), 483–492. 10.1016/j.cld.2021.03.009 34229835 PMC8996817

[B31] Hertz R. Bar-Tana J. (1998). Peroxisome Proliferator-Activated Receptor (PPAR) Alpha Activation and its Consequences in Humans. Toxicol. Lett. 102-103, 85–90. 10.1016/s0378-4274(98)00290-2 10022237

[B32] Huang L.-L. Wan J.-B. Wang B. He C.-W. Ma H. Li T.-W. (2013). Suppression of Acute Ethanol-Induced Hepatic Steatosis by Docosahexaenoic Acid Is Associated with Downregulation of Stearoyl-CoA Desaturase 1 and Inflammatory Cytokines. Prostagl. Leukot. Essent. Fat. Acids 88 (5), 347–353. 10.1016/j.plefa.2013.02.002 23474173

[B33] Huang L.-S. Yuen J. J. Trites M. J. Saha A. Epps C. T. Hu Y. (2018). Dietary Macronutrient Composition Determines the Contribution of DGAT1 to Alcoholic Steatosis. Alcohol Clin. Exp. Re 42 (12), 2298–2312. 10.1111/acer.13881 PMC6286229 30192394

[B34] Hyun J. Han J. Lee C. Yoon M. Jung Y. (2021). Pathophysiological Aspects of Alcohol Metabolism in the Liver. Int. J. Mol. Sci. 22 (11), 5717. 10.3390/ijms22115717 34071962 PMC8197869

[B35] Itabe H. Yamaguchi T. Nimura S. Sasabe N. (2017). Perilipins: a Diversity of Intracellular Lipid Droplet Proteins. Lipids Health Dis. 16 (1), 83. 10.1186/s12944-017-0473-y 28454542 PMC5410086

[B36] Jeon S. Carr R. (2020). Alcohol Effects on Hepatic Lipid Metabolism. J. Lipid Res. 61 (4), 470–479. 10.1194/jlr.r119000547 32029510 PMC7112138

[B37] Ji C. Chan C. Kaplowitz N. (2006). Predominant Role of Sterol Response Element Binding Proteins (SREBP) Lipogenic Pathways in Hepatic Steatosis in the Murine Intragastric Ethanol Feeding Model. J. Hepatology 45 (5), 717–724. 10.1016/j.jhep.2006.05.009 16879892

[B38] Kang L. Chen X. Sebastian B. M. Pratt B. T. Bederman I. R. Alexander J. C. (2007). Chronic Ethanol and Triglyceride Turnover in White Adipose Tissue in Rats. J. Biol. Chem. 282 (39), 28465–28473. 10.1074/jbc.m705503200 17686776

[B39] Keller H. Dreyer C. Medin J. Mahfoudi A. Ozato K. Wahli W. (1993). Fatty Acids and Retinoids Control Lipid Metabolism through Activation of Peroxisome Proliferator-Activated Receptor-Retinoid X Receptor Heterodimers. Proc. Natl. Acad. Sci. U.S.A. 90 (6), 2160–2164. 10.1073/pnas.90.6.2160 8384714 PMC46045

[B40] Kersten S. Seydoux J. Peters J. M. Gonzalez F. J. Desvergne B. Wahli W. (1999). Peroxisome Proliferator-Activated Receptor α Mediates the Adaptive Response to Fasting. J. Clin. Invest. 103 (11), 1489–1498. 10.1172/jci6223 10359558 PMC408372

[B41] Kharbanda K. K. Todero S. L. King A. L. Osna N. A. McVicker B. L. Tuma D. J. (2012). Betaine Treatment Attenuates Chronic Ethanol-Induced Hepatic Steatosis and Alterations to the Mitochondrial Respiratory Chain Proteome. Int. J. Hepatol. 2012, 962183. 10.1155/2012/962183 22187660 PMC3235488

[B42] Li C. Li L. Lian J. Watts R. Nelson R. Goodwin B. (2015a). Roles of Acyl-CoA:Diacylglycerol Acyltransferases 1 and 2 in Triacylglycerol Synthesis and Secretion in Primary Hepatocytes. Atvb 35 (5), 1080–1091. 10.1161/atvbaha.114.304584 25792450

[B43] Li H.-H. Tyburski J. B. Wang Y.-W. Strawn S. Moon B.-H. Kallakury B. V. S. (2014). Modulation of Fatty Acid and Bile Acid Metabolism by Peroxisome Proliferator-Activated ReceptorαProtects against Alcoholic Liver Disease. Alcohol Clin. Exp. Res. 38 (6), 1520–1531. 10.1111/acer.12424 24773203 PMC4047177

[B44] Li X. Lian F. Liu C. Hu K.-Q. Wang X.-D. (2015b). Isocaloric Pair-Fed High-Carbohydrate Diet Induced More Hepatic Steatosis and Inflammation Than High-Fat Diet Mediated by miR-34a/SIRT1 Axis in Mice. Sci. Rep. 5, 16774. 10.1038/srep16774 26608583 PMC4660435

[B45] Liangpunsakul S. Ross R. A. Crabb D. W. (2013). Activation of Carbohydrate Response Element-Binding Protein by Ethanol. J. Investig. Med. 61 (2), 270–277. 10.2310/jim.0b013e31827c2795 PMC3554838 23266705

[B46] Liangpunsakul S. Sozio M. S. Shin E. Zhao Z. Xu Y. Ross R. A. (2010). Inhibitory Effect of Ethanol on AMPK Phosphorylation Is Mediated in Part through Elevated Ceramide Levels. Am. J. Physiology-Gastrointestinal Liver Physiology 298 (6), G1004–G1012. 10.1152/ajpgi.00482.2009 PMC3774334 20224005

[B47] Lieber C. S. (1988). Biochemical and Molecular Basis of Alcohol-Induced Injury to Liver and Other Tissues. N. Engl. J. Med. 319 (25), 1639–1650. 10.1056/NEJM198812223192505 3059192

[B48] Longato L. Ripp K. Setshedi M. Dostalek M. Akhlaghi F. Branda M. (2012). Insulin Resistance, Ceramide Accumulation, and Endoplasmic Reticulum Stress in Human Chronic Alcohol-Related Liver Disease. Oxid. Med. Cell. Longev. 2012, 479348. 10.1155/2012/479348 22577490 PMC3347750

[B49] Lounis M. A. Escoula Q. Veillette C. Bergeron K.-F. Ntambi J. M. Mounier C. (2016). SCD1 Deficiency Protects Mice against Ethanol-Induced Liver Injury. Biochimica Biophysica Acta (BBA) - Mol. Cell. Biol. Lipids 1861 (11), 1662–1670. 10.1016/j.bbalip.2016.07.012 27477676

[B50] MacDonald M. L. E. Singaraja R. R. Bissada N. Ruddle P. Watts R. Karasinska J. M. (2008). Absence of Stearoyl-CoA Desaturase-1 Ameliorates Features of the Metabolic Syndrome in LDLR-Deficient Mice. J. Lipid Res. 49 (1), 217–229. 10.1194/jlr.m700478-jlr200 17960025 PMC5017869

[B51] Mak K. M. Ren C. Ponomarenko A. Cao Q. Lieber C. S. (2008). Adipose Differentiation-Related Protein Is a Reliable Lipid Droplet Marker in Alcoholic Fatty Liver of Rats. Alcohol. Clin. Exp. Res. 32 (4), 683–689. 10.1111/j.1530-0277.2008.00624.x 18341646

[B52] Mann R. E. Smart R. G. Govoni R. (2003). The Epidemiology of Alcoholic Liver Disease. Alcohol Res. Health 27 (3), 209–219. 15535449 PMC6668879

[B53] Marmier S. Dentin R. Daujat-Chavanieu M. Guillou H. Bertrand-Michel J. Gerbal-Chaloin S. (2015). Novel Role for Carbohydrate Responsive Element Binding Protein in the Control of Ethanol Metabolism and Susceptibility to Binge Drinking. Hepatology 62 (4), 1086–1100. 10.1002/hep.27778 25761756

[B54] Miller A. M. Horiguchi N. Jeong W.-I. Radaeva S. Gao B. (2011). Molecular Mechanisms of Alcoholic Liver Disease: Innate Immunity and Cytokines. Alcohol Clin. Exp. Res. 35 (5), 787–793. 10.1111/j.1530-0277.2010.01399.x 21284667 PMC3083482

[B55] Nakajima T. Kamijo Y. Tanaka N. Sugiyama E. Tanaka E. Kiyosawa K. (2004). Peroxisome Proliferator-Activated Receptor α Protects against Alcohol-Induced Liver Damage. Hepatology 40 (4), 972–980. 10.1002/hep.1840400428 15382117

[B56] Nanji A. A. Dannenberg A. J. Jokelainen K. Bass N. M. (2004). Alcoholic Liver Injury in the Rat Is Associated with Reduced Expression of Peroxisome Proliferator-α (PPARα)-Regulated Genes and Is Ameliorated by PPARα Activation. J. Pharmacol. Exp. Ther. 310 (1), 417–424. 10.1124/jpet.103.064717 15016835

[B57] Ntambi J. M. Miyazaki M. Stoehr J. P. Lan H. Kendziorski C. M. Yandell B. S. (2002). Loss of Stearoyl-CoA Desaturase-1 Function Protects Mice against Adiposity. Proc. Natl. Acad. Sci. U.S.A. 99 (17), 11482–11486. 10.1073/pnas.132384699 12177411 PMC123282

[B58] Okiyama W. Tanaka N. Nakajima T. Tanaka E. Kiyosawa K. Gonzalez F. J. (2009). Polyenephosphatidylcholine Prevents Alcoholic Liver Disease in PPARα-Null Mice through Attenuation of Increases in Oxidative Stress. J. Hepatology 50 (6), 1236–1246. 10.1016/j.jhep.2009.01.025 PMC2809859 19398233

[B59] Orlicky D. J. Roede J. R. Bales E. Greenwood C. Greenberg A. Petersen D. (2011). Chronic Ethanol Consumption in Mice Alters Hepatocyte Lipid Droplet Properties. Alcohol Clin. Exp. Res. 35 (6), 1020–1033. 10.1111/j.1530-0277.2011.01434.x 21535024 PMC4158406

[B60] Osna N. A. Donohue T. M. Jr. Kharbanda K. K. (2017). Alcoholic Liver Disease: Pathogenesis and Current Management. Alcohol Res. 38 (2), 147–161. 28988570 10.35946/arcr.v38.2.01 PMC5513682

[B61] Palmer C. N. A. Hsu M.-H. Griffin K. J. Raucy J. L. Johnson E. F. (1998). Peroxisome Proliferator Activated Receptor-α Expression in Human Liver. Mol. Pharmacol. 53 (1), 14–22. 10.1124/mol.53.1.14 9443928

[B62] Pettinelli P. Videla L. A. (2011). Up-Regulation of PPAR-γ mRNA Expression in the Liver of Obese Patients: an Additional Reinforcing Lipogenic Mechanism to SREBP-1c Induction. J. Clin. Endocrinol. Metab. 96 (5), 1424–1430. 10.1210/jc.2010-2129 21325464

[B63] Quiroga A. D. Li L. Trötzmüller M. Nelson R. Proctor S. D. Köfeler H. (2012). Deficiency of Carboxylesterase 1/esterase-X Results in Obesity, Hepatic Steatosis, and Hyperlipidemia. Hepatology 56 (6), 2188–2198. 10.1002/hep.25961 22806626

[B64] Rachakonda V. Gabbert C. Raina A. Bell L. N. Cooper S. Malik S. (2014). Serum Metabolomic Profiling in Acute Alcoholic Hepatitis Identifies Multiple Dysregulated Pathways. PLoS One 9 (12), e113860. 10.1371/journal.pone.0113860 25461442 PMC4252257

[B65] Redlich C. A. Blaner W. S. Van Bennekum A. M. Chung J. S. Clever S. L. Holm C. T. (1998). Effect of Supplementation with Beta-Carotene and Vitamin A on Lung Nutrient Levels. Cancer Epidemiol. Biomarkers Prev. 7 (3), 211–214. 9521435

[B66] Ronis M. J. J. Hennings L. Stewart B. Basnakian A. G. Apostolov E. O. Albano E. (2011). Effects of Long-Term Ethanol Administration in a Rat Total Enteral Nutrition Model of Alcoholic Liver Disease. Am. J. Physiology-Gastrointestinal Liver Physiology 300 (1), G109–G119. 10.1152/ajpgi.00145.2010 PMC3025509 21051528

[B67] Schadinger S. E. Bucher N. L. R. Schreiber B. M. Farmer S. R. (2005). PPARγ2 Regulates Lipogenesis and Lipid Accumulation in Steatotic Hepatocytes. Am. J. Physiology-Endocrinology Metabolism 288 (6), E1195–E1205. 10.1152/ajpendo.00513.2004 15644454

[B68] Shaheen A. A. Kong K. Ma C. Doktorchik C. Coffin C. S. Swain M. G. (2021). Impact of the COVID-19 Pandemic on Hospitalizations for Alcoholic Hepatitis or Cirrhosis in Alberta, Canada. Clin. Gastroenterol. Hepatol. 20, e1170–e1179. 10.1016/j.cgh.2021.10.030 34715379 PMC8547973

[B69] Shen H. Jiang L. Lin J. D. Omary M. B. Rui L. (2019). Brown Fat Activation Mitigates Alcohol-Induced Liver Steatosis and Injury in Mice. J. Clin. Invest. 129 (6), 2305–2317. 10.1172/jci124376 30888335 PMC6546460

[B70] Sherlock S. Dooley J. (2008). Diseases of the Liver and Biliary System. Hoboken: Wiley-Blackwell.

[B71] Siler S. Q. Neese R. A. Hellerstein M. K. (1999). De Novo lipogenesis, Lipid Kinetics, and Whole-Body Lipid Balances in Humans after Acute Alcohol Consumption. Am. J. Clin. Nutr. 70 (5), 928–936. 10.1093/ajcn/70.5.928 10539756

[B72] Singal A. K. Shah V. H. (2019). Current Trials and Novel Therapeutic Targets for Alcoholic Hepatitis. J. Hepatology 70 (2), 305–313. 10.1016/j.jhep.2018.10.026 30658731

[B73] Sohal A. Khalid S. Green V. Gulati A. Roytman M. (2021). The Pandemic within the Pandemic: Unprecedented Rise in Alcohol-Related Hepatitis during the COVID-19 Pandemic. J. Clin. Gastroenterol. 56, e171–e175. 10.1097/MCG.0000000000001627 PMC8843054 34653062

[B74] Stahl A. Gimeno R. E. Tartaglia L. A. Lodish H. F. (2001). Fatty Acid Transport Proteins: a Current View of a Growing Family. Trends Endocrinol. Metab. 12 (6), 266–273. 10.1016/s1043-2760(01)00427-1 11445444

[B75] Stockwell T. Andreasson S. Cherpitel C. Chikritzhs T. Dangardt F. Holder H. (2021). The Burden of Alcohol on Health Care during COVID ‐19. Drug Alcohol Rev. 40 (1), 3–7. 10.1111/dar.13143 32835427 PMC7461236

[B76] Stone S. J. Myers H. M. Watkins S. M. Brown B. E. Feingold K. R. Elias P. M. (2004). Lipopenia and Skin Barrier Abnormalities in DGAT2-Deficient Mice. J. Biol. Chem. 279 (12), 11767–11776. 10.1074/jbc.m311000200 14668353

[B77] Straub B. K. Stoeffel P. Heid H. Zimbelmann R. Schirmacher P. (2008). Differential Pattern of Lipid Droplet-Associated Proteins Andde Novoperilipin Expression in Hepatocyte Steatogenesis. Hepatology 47 (6), 1936–1946. 10.1002/hep.22268 18393390

[B78] Stumvoll M. Wahl H. G. Löblein K. Becker R. Volk A. Renn W. (2001). A Novel Use of the Hyperinsulinemic-Euglycemic Clamp Technique to Estimate Insulin Sensitivity of Systemic Lipolysis. Horm. Metab. Res. 33 (2), 89–95. 10.1055/s-2001-12403 11294499

[B79] Sugimoto T. Yamashita S. Ishigami M. Sakai N. Hirano K.-i. Tahara M. (2002). Decreased Microsomal Triglyceride Transfer Protein Activity Contributes to Initiation of Alcoholic Liver Steatosis in Rats. J. Hepatology 36 (2), 157–162. 10.1016/s0168-8278(01)00263-x 11830326

[B80] Tomita K. Azuma T. Kitamura N. Nishida J. Tamiya G. Oka A. (2004). Pioglitazone Prevents Alcohol-Induced Fatty Liver in Rats through Up-Regulation of C-Met. Gastroenterology 126 (3), 873–885. 10.1053/j.gastro.2003.12.008 14988841

[B81] Tugwood J. D. Aldridge T. C. Lambe K. G. Macdonald N. Woodyatt N. J. (1996). Peroxisome Proliferator-Activated Receptors: Stuctures and Function. Ann. N. Y. Acad. Sci. 804, 252–265. 10.1111/j.1749-6632.1996.tb18620.x 8993548

[B82] Wada S. Yamazaki T. Kawano Y. Miura S. Ezaki O. (2008). Fish Oil Fed Prior to Ethanol Administration Prevents Acute Ethanol-Induced Fatty Liver in Mice. J. Hepatology 49 (3), 441–450. 10.1016/j.jhep.2008.04.026 18620774

[B83] Wan Y. J. Morimoto M. Thurman R. G. Bojes H. K. French S. W. (1995). Expression of the Peroxisome Proliferator-Activated Receptor Gene Is Decreased in Experimental Alcoholic Liver Disease. Life Sci. 56 (5), 307–317. 10.1016/0024-3205(94)00953-8 7837930

[B84] Wang Z. Yao T. Song Z. (2010). Involvement and Mechanism of DGAT2 Upregulation in the Pathogenesis of Alcoholic Fatty Liver Disease. J. Lipid Res. 51 (11), 3158–3165. 10.1194/jlr.m007948 20739640 PMC2952556

[B85] Wei X. Shi X. Zhong W. Zhao Y. Tang Y. Sun W. (2013). Chronic Alcohol Exposure Disturbs Lipid Homeostasis at the Adipose Tissue-Liver axis in Mice: Analysis of Triacylglycerols Using High-Resolution Mass Spectrometry in Combination with *In Vivo* Metabolite Deuterium Labeling. PLoS One 8 (2), e55382. 10.1371/journal.pone.0055382 23405143 PMC3566154

[B86] WHO (2018). Global Status Report on Alcohol and Health 2018. AvaliableAt: https://www.who.int/publications-detail-redirect/9789241565639 (Accessed November 03, 2021).

[B87] Wurie H. R. Buckett L. Zammit V. A. (2012). Diacylglycerol Acyltransferase 2 Acts Upstream of Diacylglycerol Acyltransferase 1 and Utilizes Nascent Diglycerides Andde Novosynthesized Fatty Acids in HepG2 Cells. FEBS J. 279 (17), 3033–3047. 10.1111/j.1742-4658.2012.08684.x 22748069

[B88] Xu J. Li Y. Chen W.-D. Xu Y. Yin L. Ge X. (2014). Hepatic Carboxylesterase 1 Is Essential for Both Normal and Farnesoid X Receptor-Controlled Lipid Homeostasis. Hepatology 59 (5), 1761–1771. 10.1002/hep.26714 24038130 PMC3938573

[B89] Xu J. Xu Y. Li Y. Jadhav K. You M. Yin L. (2016). Carboxylesterase 1 Is Regulated by Hepatocyte Nuclear Factor 4α and Protects against Alcohol- and MCD Diet-Induced Liver Injury. Sci. Rep. 6, 24277. 10.1038/srep24277 27075303 PMC4831009

[B90] Xue M. Liang H. Zhou Z. Liu Y. He X. Zhang Z. (2021). Effect of Fucoidan on Ethanol-Induced Liver Injury and Steatosis in Mice and the Underlying Mechanism. Food Nutr. Res. 65, 5384. 10.29219/fnr.v65.5384 PMC8098649 33994911

[B91] Yamashita H. Kaneyuki T. Tagawa K. (2001). Production of Acetate in the Liver and its Utilization in Peripheral Tissues. Biochim. Biophys. Acta 1532 (1-2), 79–87. 10.1016/s1388-1981(01)00117-2 11420176

[B92] Yin H.-Q. Kim M. Kim J.-H. Kong G. Kang K.-S. Kim H.-L. (2007). Differential Gene Expression and Lipid Metabolism in Fatty Liver Induced by Acute Ethanol Treatment in Mice. Toxicol. Appl. Pharmacol. 223 (3), 225–233. 10.1016/j.taap.2007.06.018 17655900

[B93] You M. Arteel G. E. (2019). Effect of Ethanol on Lipid Metabolism. J. Hepatology 70 (2), 237–248. 10.1016/j.jhep.2018.10.037 PMC6436537 30658725

[B94] You M. Fischer M. Deeg M. A. Crabb D. W. (2002). Ethanol Induces Fatty Acid Synthesis Pathways by Activation of Sterol Regulatory Element-Binding Protein (SREBP). J. Biol. Chem. 277 (32), 29342–29347. 10.1074/jbc.m202411200 12036955

[B95] Yu J. H. Song S. J. Kim A. Choi Y. Seok J. W. Kim H. J. (2016). Suppression of PPARγ-Mediated Monoacylglycerol O-Acyltransferase 1 Expression Ameliorates Alcoholic Hepatic Steatosis. Sci. Rep. 6, 29352. 10.1038/srep29352 27404390 PMC4941543

[B96] Yu S. Rao S. Reddy J. K. (2003). Peroxisome Proliferator-Activated Receptors, Fatty Acid Oxidation, Steatohepatitis and Hepatocarcinogenesis. Curr. Mol. Med. 3 (6), 561–572. 10.2174/1566524033479537 14527087

[B97] Zeng H. Qin H. Liao M. Zheng E. Luo X. Xiao A. (2022). CD36 Promotes De Novo Lipogenesis in Hepatocytes through INSIG2-dependent SREBP1 Processing. Mol. Metab. 57, 101428. 10.1016/j.molmet.2021.101428 34974159 PMC8810570

[B98] Zhang M. Wang C. Wang C. Zhao H. Zhao C. Chen Y. (2015). Enhanced AMPK Phosphorylation Contributes to the Beneficial Effects of Lactobacillus Rhamnosus GG Supernatant on Chronic-Alcohol-Induced Fatty Liver Disease. J. Nutr. Biochem. 26 (4), 337–344. 10.1016/j.jnutbio.2014.10.016 25622859 PMC6679353

[B99] Zhang N. Hu Y. Ding C. Zeng W. Shan W. Fan H. (2017). Salvianolic Acid B Protects against Chronic Alcoholic Liver Injury via SIRT1-Mediated Inhibition of CRP and ChREBP in Rats. Toxicol. Lett. 267, 1–10. 10.1016/j.toxlet.2016.12.010 27989594

[B100] Zhang W. Sun Q. Zhong W. Sun X. Zhou Z. (2016). Hepatic Peroxisome Proliferator-Activated Receptor Gamma Signaling Contributes to Alcohol-Induced Hepatic Steatosis and Inflammation in Mice. Alcohol Clin. Exp. Res. 40 (5), 988–999. 10.1111/acer.13049 27062444 PMC5742869

[B101] Zhong W. Zhao Y. Tang Y. Wei X. Shi X. Sun W. (2012). Chronic Alcohol Exposure Stimulates Adipose Tissue Lipolysis in Mice. Am. J. Pathology 180 (3), 998–1007. 10.1016/j.ajpath.2011.11.017 PMC3349880 22234172

